# Individualized Web-Based Exercise for the Treatment of Depression: Randomized Controlled Trial

**DOI:** 10.2196/10698

**Published:** 2018-10-12

**Authors:** Nils Haller, Sonja Lorenz, Daniel Pfirrmann, Cora Koch, Klaus Lieb, Ulrich Dettweiler, Perikles Simon, Patrick Jung

**Affiliations:** 1 Department of Sports Medicine, Rehabilitation and Disease Prevention Johannes Gutenberg-University Mainz Germany; 2 Department of Psychiatry and Psychotherapy University Medical Center Mainz Germany; 3 Faculty of Arts and Education University of Stavanger Stavanger Norway

**Keywords:** depression, exercise, Web-based intervention, eHealth

## Abstract

**Background:**

Due to the high prevalence of depressive disorders, it is mandatory to develop therapeutic strategies that provide universal access and require limited financial and human resources. Web-based therapeutic approaches fulfill these conditions.

**Objective:**

The objective of our study was to assess the feasibility, acceptability, and efficacy of a supervised, individualized 8-week Web-based exercise intervention conducted for patients with moderate to severe depression.

**Methods:**

We recruited 20 patients with unipolar depression and randomly assigned them into 2 groups (intervention, exercise program group, n=14, and control, treatment-as-usual group, n=6). At baseline, depressive symptoms were rated via the Quick Inventory of Depressive Symptomatology (QIDS) by patients themselves (QIDS–self-report, QIDS-SR) and by a blinded psychiatrist (QIDS–clinician rating, QIDS-C). In addition, performance diagnostics (lactate analysis, spiroergometry during a treadmill walking test) were conducted. Quality of life was assessed via the Short Form-36 questionnaire (SF-36) and self-efficacy via the General Self-Efficacy scale (GSE). In addition, habitual physical activity (HPA) was determined via the Baecke questionnaire. Participants of the intervention group received exercise schedules once weekly with endurance and strength training instructions. Rating of depressive symptoms was repeated after 6-12 days and 8 weeks; performance diagnostics and the completion of all the questionnaires were repeated after 8 weeks only.

**Results:**

The severity of depression subsided significantly in the intervention group after 8 weeks (median change in QIDS-SR: −5; interquartile range, IQR: −2 to −10), although it was already evident within the first 6-12 days (median change in QIDS-SR: −6; IQR: −2 to −8). During the intervention, participants undertook a median of 75 (IQR: 63 to 98) minutes of endurance training per week or 84% (16 [IQR: 9 to 19] of 19 [IQR: 15 to 21]) recommended endurance units in total. In addition, 9 (IQR: 4 to 12) of 10 (IQR: 8 to 13) recommended strength training exercise units were conducted during the 8 weeks. Performance diagnostics revealed a substantial increase in the maximum output in Watt for the intervention group after 8 weeks. Moreover, the intervention showed a favorable effect on SF-36 items “emotional well-being” and “social functioning” as well as on GSE and HPA scores.

**Conclusions:**

Our individualized Web-based exercise intervention for moderate to severe depression was highly accepted by the patients and led to a significant and clinically relevant improvement of depressive symptoms.

**Trial Registration:**

ClinicalTrials.gov NCT02874833; https://clinicaltrials.gov/ct2/show/NCT02874833 (Archived by WebCite at http://www.webcitation.org/72ZUUR4tE)

## Introduction

Unipolar depression or major depressive disorder (MDD) is the worldwide leading cause of disability [1] with a life time prevalence of around 17% [2]. MDD is commonly treated with either antidepressive medication or psychotherapy or both [3]. However, side effects of and an often skeptical attitude toward pharmacotherapy lead to poor compliance [4]. In addition, about 30%-50% of patients do not respond adequately to antidepressants [5,6]. On the other hand, personalized psychotherapy requires high personal effort, especially for highly prevalent diseases such as MDD. However, such high personnel expenses cannot be implemented, especially in rural areas. The resulting imbalance of supply and demand leads to waiting periods of several months [7]. Thus, the development of easily accessible, cost-saving, ubiquitous, and effective treatment strategies, well accepted by patients, is of great importance in health politics. In this study, we exploited such an innovative form of therapy in terms of an individualized, supervised, Web-based exercise therapy in patients with MDD and moderate to severe depressive symptoms. We chose this approach because physical activity is currently recommended as a safe and effective adjunctive therapy in the treatment of MDD in numerous national guidelines [8-10].

To the best of our knowledge, this is the first Web-based exercise approach in the treatment of MDD. Previous Web-based trials on depression have focused on self-help and cognitive behavioral therapy (CBT) [11-13]. Meta-analyses assessing the effectiveness of these interventions on depression severity have reported variable results [14,15]. However, moderate effects on depressive symptoms were reported when Web-based CBT was offered in an individually tailored form [16]. Accordingly, our Web-based exercise program was monitored and individually adapted for each patient.

So far, Web-based exercise therapy has been applied to a variety of patient groups such as type 2 diabetes patients [17,18], breast cancer survivors [19], or osteoarthritis patients [20]. The outcomes in the respective studies were promising in terms of improved blood sugar parameters [18], reduced fatigue [19], or an enhanced activity level [18-20]. Thus, Web-based exercise interventions have a remarkable potential for improving health-related outcomes.

In the last years, exercise has garnered growing interest as a component in the treatment of depression. Evidence suggests that exercise may lead to a marked reduction in depressive symptoms, comparable with pharmacotherapy, after 16 weeks [5,21]. The latest Cochrane Review [22] reported a moderate clinical effect of exercise on depressive symptoms. However, if only studies with high methodological quality were considered, the effects of exercise were shown to be only small to moderate, in line with the meta-analysis of randomized controlled trials by Krogh et al [23] and Josefsson et al [3]. In contrast, a more recent meta-analysis supported the assumption of an underestimation of exercise effects on depressive symptoms due to publication bias [24]. In sum, further standardized studies are needed to give a robust estimate about the therapeutic effect size of exercise.

It has not yet been clarified which type, duration, and intensity of exercise is most effective in depression [22] and whether there are exercise-specific physiological changes that mediate antidepressive effects [25]. The specific psychological or biological mechanisms through which physical activity may lead to positive effects on depressive symptoms remain a matter of current research. However, exercise seems to be a promising adjunctive therapy option for depression, given that there exists no specific monomodal therapy that is effective in every patient [21].

In contrast to previous exercise studies on depression [5,26,27], we developed an individualized Web-based approach that did not schedule attendance exercise sessions. The purpose of this study is to evaluate (1) the feasibility and (2) the antidepressive effects of our Web-based exercise program.

## Methods

### General Information and Ethics

The multidisciplinary single-center trial was a collaboration between the Department of Psychiatry and Psychotherapy and the Institute of Sports Medicine of the University of Mainz. The study was designed as a feasibility study, which would be continued with higher sample sizes and under participation of multiple centers if the study provided promising outcomes. All procedures were approved by the regional Ethical Board Mainz, Germany. Previous and ongoing treatment was not affected by study participation.

### Inclusion Criteria

Patients who fulfilled the following criteria were included in the study:

1. Ability to understand the purpose and risks of the study and provide signed and dated informed consent and authorization to use confidential health information in accordance with national and local subject privacy regulations
2. Sufficient computer or internet literacy to get along with our internet platform
3. Aged 20-65 years, inclusive, at the time of informed consent
4. Montreal Cognitive Assessment [28] >18 to exclude moderate to severe cognitive impairment
5. Apart from a clinical diagnosis of major depression or bipolar affective disorder, the subject must be in good health as determined by the investigator based on medical history and physical examination.
6. Quick Inventory of Depressive Symptomatology (QIDS) scores >5
7. No changes in antidepressive therapy in the 4 weeks before study entry

For detailed exclusion criteria, please refer to ClinicalTrials.gov (NCT02874833).

### Participants and Randomization

Participants were recruited offline between July 2016 and October 2017 through local outpatient psychiatrists. Two patients disclaimed our offer to participate in the study. A total of 20 participants with depressive symptoms were enrolled after 2 of 22 patients were excluded due to myocardial inflammation and pregnancy. Patients did not receive any compensation for participating in the study.

**Figure 1:**
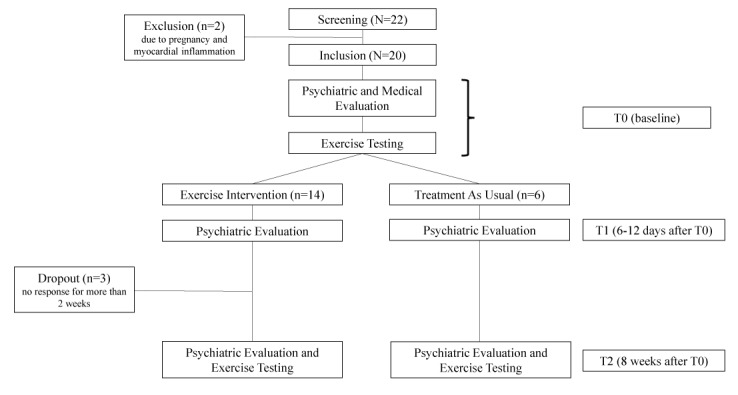
Flowchart of the study.

For randomization, numbers between 0 and 1 were randomly computer generated and used for assignment to either the control group (values ≤.3) or intervention group (IG; values of >.3). At baseline (T0), depressive symptoms were rated by patients themselves and by a blinded psychiatrist (SL or CK). Thereafter, patients were subjected to performance diagnostics (lactate diagnostics, spiroergometry), which was followed by either an 8-week supervised, individualized Web-based exercise program (IG) or treatment as usual (control group). Within 6-12 (median: 9) days after T0, the rating was repeated (T1). The final examination, including clinical rating and performance diagnostics, took place after 8 weeks (T2). Controls underwent all examinations, while any other form of existing therapy (eg, antidepressive medication) was not affected (Figure 1).

### Intervention and Internet Platform

**Figure 2:**
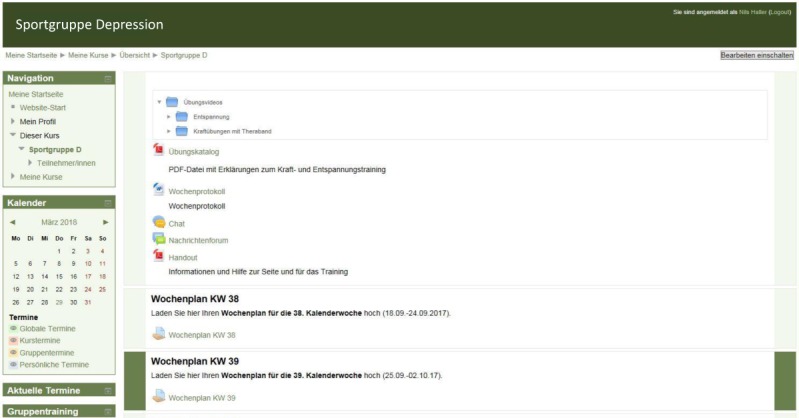
Design of our home page with chat and message function, training videos, and upload area for training schedules.

IG patients gained access to our home page (Figure 2) and were provided with a heart rate monitor (Polar FT1; Polar Electro, Büttelborn, Germany) and 4 different types of resistance bands (Thera-Band, Akron, OH, United States). The platform was designed to be user friendly [29]. Message function was used to send exercise schedules to the patients once weekly. After each week, motivational feedback was given to improve adherence [30,31].

Schedules included the recommended extent of exercise with a maximum of 3 endurance and 2 strength training units per week. An additional group training session was offered biweekly by a sports therapist. At the end of each week, patients were expected to upload a protocol of their weekly activity on our platform, making the protocol available to the supervisor. Based on this response, training goals were individually adapted in the duration and intensity for the following week to keep motivation high and prevent patients from overload and frustration.

### Structure of Weekly Exercise

**Figure 3:**
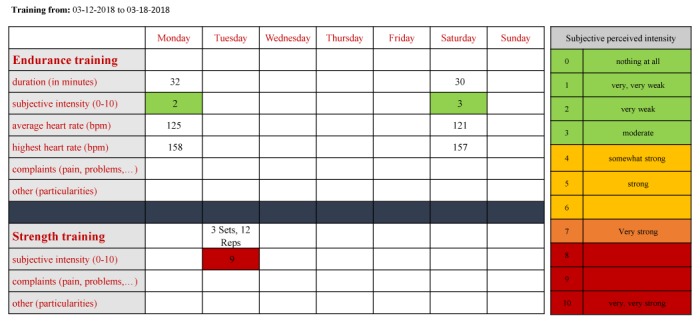
Activity protocol of one participant; patients responded after each week by uploading their filled protocol either as a scan or a Word document. In this example, intensity and duration of the endurance training was recommend to be increased, whereas the intensity of the strength training was advised to be decreased.

Endurance exercise recommendations were based on heart rate (baseline +1.5 mmol model [32]) with a duration of 30-60 minutes per unit; this has been proven to be effective in the reduction of depressive symptoms [5,27,30]. However, as suggested by Craft and Landers [33] and in line with guidelines [34], it was necessary for some untrained or unexperienced subjects to start with a more moderate duration of 20 minutes per session and 2 units per week. Patients with a poor exercise capacity were recommended to start with walking instead of jogging. By taking these personal preferences and individual conditions into account, we aimed at achieving high adherence [27,30]. Furthermore, adjustment and weekly progression of the endurance training were assessed via Borg scale [35] (Figure 3) to keep the intensity in a moderate to vigorous range according to common guidelines and recommendations [8,9,30].

If patients reported Borg values to be <4, training intensity was moderately increased [36] in terms of an expanded duration of approximately 10 minutes per week or by recommending a higher average heart rate, leading to a higher intensity. In case of fatigue or injury or if exercise was too hard (Borg>7), intensity and duration were reduced according to patients’ request. In this case, alternative units such as relaxing were recommended. Furthermore, strength training exercises for major muscle groups were performed at home following the detailed instructions provided on our home page. Progression was ensured with increased sets and repetitions or by changing the type of resistance band.

### Evaluation of Primary Outcomes

#### Adherence

Adherence has been defined as attendance, lack of dropout, participation rate, or the fulfillment of predefined goals [37]. IG patients dropped out if no protocol was uploaded for >2 weeks. Controls, however, dropped out if they failed to attend the final examination at T2 [38]. The participation rate in our exercise program was defined as (1) training units conducted and (2) the ratio of training units conducted and training units recommended [21,38]. Data were extracted from the weekly protocols of participants (Figure 2). All units that did not count to regular, prescribed strength or endurance exercise, such as hiking or relaxing, were counted as alternative units. In addition, after T2, using a self-developed questionnaire among patients, we evaluated satisfaction with our program and reasons for not meeting our exercise recommendations.

#### Depression Scales

The severity of depressive symptoms was determined using QIDS (clinician rating, QIDS-C, and self-report, QIDS-SR) [39]. Rating was conducted at baseline, at T1 within 6-12 days, and after 8 weeks. QIDS scores can range from 0 to a maximum of 36. QIDS showed good internal consistency (Cronbach alpha=.86), and QIDS-SR16 total scores highly correlated with IDS-SR30 (*r*=.96) and HAM-D24 (*r*=.86; [39]).

### Evaluation of Secondary Outcomes

#### Self-Efficacy, Quality of Life, and Habitual Physical Activity

The Short Form-36 (SF-36) Quality of Life questionnaire [40] was filled out by every patient at baseline and after 8 weeks. SF-36 items show high reliability (Cronbach alpha>.85, reliability coefficient>.75) and construct validity [41]. In addition, the General Self-Efficacy scale (GSE) [42] and habitual physical activity (HPA) [43] were assessed at T0 and T2.

#### Physiological Parameters

After clinical rating and answering questionnaires, all patients performed a treadmill walking test until exhaustion to determine peak oxygen uptake (VO_2_ peak) and lactate threshold. Slope and velocity were increased stepwise after 3 minutes of walking at a time, as described elsewhere [36]. Lactate samples were drawn after every step to determine lactate threshold using the baseline +1.5 mmol model [32]. Maximum output in Watt was determined using the formula: 9.81 × weight (kg) × velocity (m/s) × sin α.

### Statistical Methods

For statistical analysis, we used JMP13 (SAS, Cary, NC, USA), SPSS 23 (IBM, Chicago, IL, USA), and the software package R, version 3.4.2 (2017-09-28, R Foundation for Statistical Computing, Vienna, Austria). Differences in baseline characteristics were examined using Mann-Whitney U test. The correlation between QIDS-SR and QIDS-C was calculated using Spearman’s rank correlation coefficient. In addition, comparisons at different time-points were determined using Wilcoxon signed-rank test and Bonferroni correction for multiple testing. To evaluate the influence of the intervention compared with that of the control condition, analysis of covariance (ANCOVA) was performed for each outcome. The outcomes at T2 served as dependent variables and the ones at T0 as covariates. To exclude a violation of ANCOVA preconditions, we tested for normal distribution in the residuals of the dependent variable and for homogeneity of regression slopes by analyzing the interaction term of independent variable × covariate. Furthermore, *P*<.05 was considered as statistically significant.

For the explorative analysis of the trajectories of the QIDS-SR and QIDS-C measures, we fit multilevel linear models. Hereby, the number of training units in the actual number of days in the program after T0 was used as covariates, and the observations for individuals were nested in the respective groups, that is, intervention or control. Furthermore, the log-linear transformation of the response variables QIDS-SR and QIDS-C showed better model fit, hinting at an exponential decay over time. For the covariate analysis, missing data were dealt with by using the last observation carried forward method. In the explorative multilevel analysis, missing data were omitted from the dataset.

## Results

### Patients’ Characteristics

All patients included were diagnosed with major depression, none with bipolar affective disorder. Of all, 14 patients were assigned to the IG, while 6 patients served as controls. Table 1 outlines the clinical characteristics of the patients at baseline. Of note, 15% (3/20) patients dropped out after T1 and before T2, which were all IG patients, while all 6 controls completed the study. Dropouts were because of missing responses for >2 weeks after T1 (Figure 1). Medication did not change over the course of the study, except for 1 IG subject for whom a dosage reduction of an antidepressive drug was prescribed by the treating physician.

At baseline, patients rated their depressive symptoms with a mean QIDS-SR score of 16, that is, severe depressive symptoms, whereas QIDS-C was rated slightly lower at 14, representing moderate depressive symptoms [39]. QIDS-SR and QIDS-C scores were correlated with each other (*r*=.64, *P*<.001). The mean physical fitness in terms of VO_2_ peak was determined at 26 mL/min/kg, which matches the predicted values of healthy subjects, adjusted for sex, age, and weight [44].

### Adherence

The dropout rate for IG was 21% (3/14), while all controls completed the study. In the IG, a median of 75 (interquartile range, IQR: 63 to 98) minutes of endurance exercise was performed per week. Overall, 84% (16 [IQR: 9 to 19] of 19 [IQR: 15 to 21]) recommended endurance units were completed. In addition, patients undertook 90% (9 [IQR: 4 to 12] of 10 [IQR 8 to 13]) recommended strength training units during the intervention. Four (IQR: 2 to 28) optional, alternative training units, such as relaxing, hiking, or yoga, were furthermore executed during 8 weeks. The offer to join group training with a sports therapist was accepted by 1 patient only.

Self-reported reasons for missing the prescribed training were as follows: orthopedic problems (n=4), depressive symptoms (n=4), illness (n=3), or work (n=2). Moreover, our questionnaire revealed that 9 of 11 patients did not fear any injury, all of the 11 patients saw no risk during exercise, 9 of 11 patients perceived communication through our platform as “personal,” and 10 of 11 patients assessed the number of weekly training instructions as adequate.

### Effects on Depressive Symptoms After 8 Weeks

**Figure 4:**
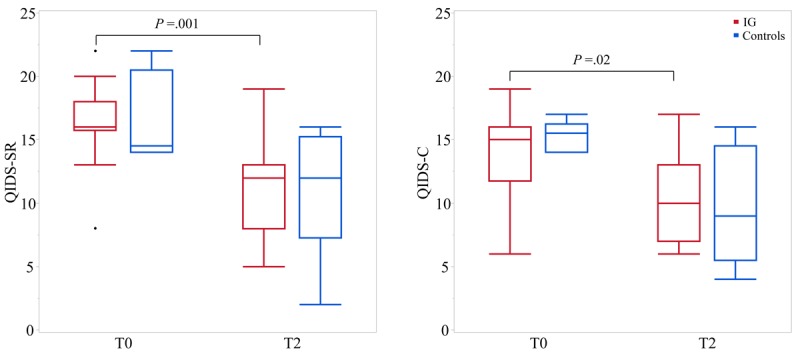
Depression scores of the controls and intervention group (IG) on the Quick Inventory of Depressive Symptomatology-self-reported (QIDS-SR; left) and QIDS-clinician rating (QIDS-C; right) at baseline (T0) and after 8 weeks (T2).

Figure 4 outlines scores of the depression scales QIDS-SR und QIDS-C at T0 and T2 in both groups. Depressive symptoms significantly decreased in IG during the 8-week intervention, as reflected in both QIDS-SR (median change: −5; IQR: −2 to −10; *P*=.001) and QIDS-C (median change: −5; IQR: −2 to −7; *P*=.02) scores. However, symptom relief was not different to controls. A reduction in depressive symptoms of ≥50% was shown via QIDS-SR in 36% (5/14) IG patients and via QIDS-C in 21% (3/14) IG patients. ANCOVA revealed no statistically significant difference between the IG and control group. However, 1 patient in the control group showed a striking improvement in depressive symptoms from a QIDS-SR score of 22 at baseline to 2 at T2.

**Table 1:** Patients’ characteristics at baseline.

Characteristics			Exercise group	Controls		Total
**Gender, n (%)**						
	Male	4 (29)		3 (50)	7 (35)	
	Female	10 (71)		3 (50)	13 (65)	
Age (years), mean (SD)			43 (14)	51 (12)		45 (14)
Height (cm), mean (SD)			171 (8)	168 (7)		170 (8)
Weight (kg), mean (SD)			77 (17)	88 (11)		80 (16)
Body mass index (kg/m^2^), mean (SD)			26.5 (5.6)	31.3 (4.3)		27.0 (5.6)
**Depression scores, mean (SD)**						
	QIDS-SR^a^	16 (3)		17 (4)	16 (3)	
	QIDS-C^b^	14 (3)		15 (1)	14 (3)	
Montreal Cognitive Assessment, mean (SD)			26 (2)	26 (3)		26 (2)
**Spiroergometry, mean (SD)**						
	VO_2_ peak^c^ (mL/min/kg)	27.0 (7.6)		23.8 (5.9)	26.0 (7.1)	
	Maximum output (Watt)	112 (37)		110 (55)	112 (42)	
**Treatment at study entry, n (%)**						
	Psychotherapy alone	2 (14)		0 (0)	2 (10)	
	Psychopharmacotherapy alone	7 (50)		3 (50)	10 (50)	
	Psychotherapy and psychopharmacotherapy	3 (21)		3 (50)	6 (30)	

^a^QIDS-SR: Quick Inventory of Depressive Symptomatology-self-reported.

^b^QIDS-C: Quick Inventory of Depressive Symptomatology-clinician-rating.

^c^VO_2_ peak: peak oxygen uptake.

**Figure 5:**
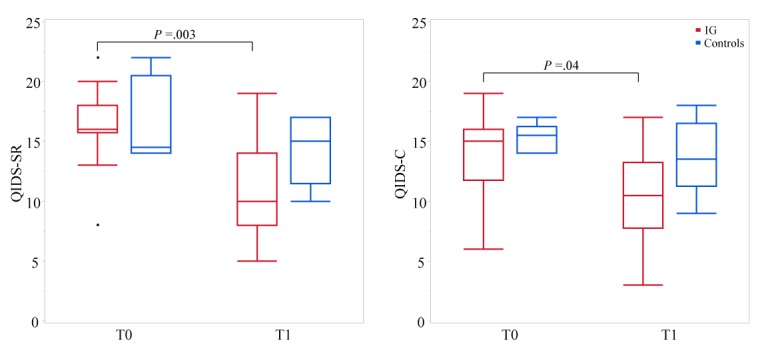
Depression scores of the controls and intervention group (IG) on the Quick Inventory of Depressive Symptomatology-self-reported (QIDS-SR; left) and QIDS-clinician rating (QIDS-C; right) at baseline (T0) and after 6-12 days (T1; median: 9 days).

### Early Antidepressive Response

Figure 5 shows depression scores of T1 after 6-12 days compared with the baseline. Depressive symptoms in IG were significantly reduced, as reflected in both QIDS-SR (median change: −6; IQR: −2 to −8; *P*=.003) and QIDS-C (median change: −3; IQR: −2 to −6; *P*=.04) scores. ANCOVA showed an estimated advantage for IG at T1 in the QIDS-SR score of −3.1 points (coefficient B). However, this effect was not statistically significant (*P*=.06; Eta², effect size=0.2). An early response of >50% reduction was observed for 36% (5/14) patients in QIDS-SR scores and for 21% (3/14) patients in QIDS-C scores. A depressive symptom reduction of ≥20% [45] was observed for 71% (10/14) patients in QIDS-SR scores and for 50% (7/14) patients in QIDS-C scores, while only 33% (2/6) controls showed an early response of ≥20% in both the QIDS-SR and QIDS-C scores.

### Self-Efficacy, Quality of Life, and Habitual Physical Activity

**Figure 6:**
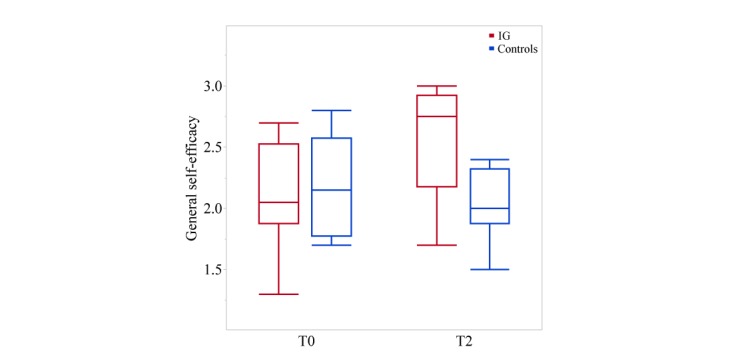
General self-efficacy scores at T0 (baseline) and T2 (after 8 weeks). Analysis of covariance revealed a favorable effect on the General Self-Efficacy scale for the intervention group (IG) compared with the controls (*P*=.02, Eta²=0.28).

Figure 6 shows the effect of the intervention and treatment as usual on GSE. ANCOVA revealed a positive influence of the intervention on SF-36 quality-of-life items “emotional well-being” (*P*=.02, Eta^2^=0.29) and “social functioning” (*P*=.04, Eta^2^=0.23), while the total quality-of-life score (*P*=.07) and the item “mental health” (*P*=.08) showed an estimated advantage. As expected, IG showed a higher level of physical activity than controls, reflected in total HPA (*P*=.007, Eta^2^=0.36) and HPA items “leisure time” (*P*=.02, Eta^2^=0.27) and “sport” (*P*=.001, Eta^2^=0.51).

### Effects on Physiological Performance Parameters

**Figure 7:**
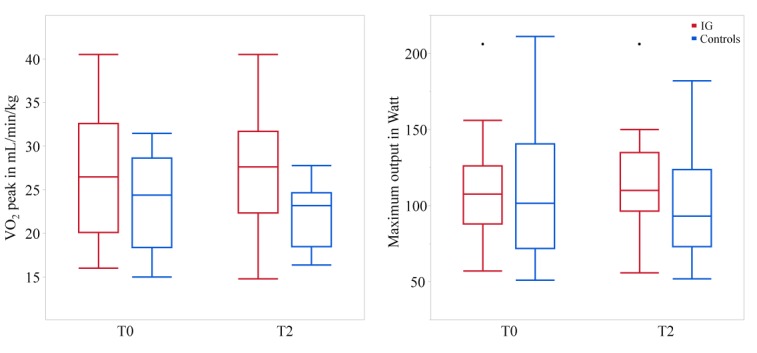
Peak oxygen uptake (VO2 peak; left) and maximum output in Watt (right) at baseline (T0) and after 8 weeks (T2) for the intervention group (IG) and the controls.

Figure 7 illustrates the physiological parameters in terms of VO_2_ peak and maximum output in Watt. IG patients showed an improvement in VO_2_ peak of 0.7 mL/min/kg while VO_2_ peak of controls declined by 1.6 mL/min/kg. The maximum output in Watt was improved by 4% in the IG, while that in controls declined by 9% after 8 weeks. Compared with controls, ANCOVA revealed no statistically significant influence on the IG regarding VO_2_ peak (*P*=.07) and lactate threshold (*P*=.09), but showed a significant influence on the maximum output in Watt (*P*=.006; Eta^2^=0.37; *B*=14.1).

### Explorative Multilevel Analysis

With respect to QIDS-SR, the multilevel analysis showed a statistically significant effect of day of the measure (estimate: −0.01 on the log-linear scale, *P*<.01) and number of training units (estimate: −0.07 on the log-linear scale, *P*=.05) as well as their interaction term (estimate: 0.001 on the log-linear scale, *P*=.04). Similar effects could be determined for QIDS-C with respect to the day of measure (estimate: −0.01 on the log-linear scale, *P*<.01) and its interaction term with training units (estimate: 0.001 on the log-linear scale, *P*=.05). The number of training units did not show a statistically significant effect on QIDS-C (*P*=.07).

### Side Effects of the Intervention

Beside minor orthopedic problems in 4 cases, no side effects of regular exercise were reported by the patients.

## Discussion

### Principal Findings

Due to the high prevalence, high personnel expenses, and therapeutic costs of MDD, it is desirable to have therapeutic strategies at hand that are effective and provide universal access with limited financial and human resource requirements. Web-based and computerized therapeutic approaches have been found to be promising to fulfill these claims [46]. To date, the focus has been on CBT [11,47] or self-help [12] internet therapy. Exercise has been suggested as an augmentation or alternative therapy for depressed patients [9,22]. Here we show for the first time that an 8-week Web-based exercise intervention with individualized weekly training schedules is feasible and effective in patients with moderate to severe depressive symptoms.

### Adherence

During the 8-week intervention, patients conducted 84% (16/19) of recommended endurance units and 90% (9/10) strength training units, which exceeds [48] or is comparable to the adherence reported in previous studies regarding attendance [5,27] or home-based exercise, that is, exercising at home with regular contact to the supervisor [21].

The depression severity in this study was moderate to severe, that is, higher than that in previous trials that investigated the effects of exercise on depressive symptoms [21,26,27]. Christensen et al [38] reported that the probability of adherence in Web-based therapeutic trials decreases with the severity of depression. It is, thus, somewhat surprising that adherence in this study was comparable to that in previous ones [5,21,27]. The dropout rate in our study was 21% in the IG, comparable to personalized psychotherapy [49] but probably lower than that for psychotropic drugs. Up to 50% do not adhere to their antidepressants [50].

The high therapeutic adherence in our study might partially be attributed to the preferential recruitment of patients with a relatively high interest in sports. Our patient sample reached the predicted VO_2_ peak values [44], in contrast to the expected lower physical fitness values in depression [51]. This suggests that the physical fitness of our participants was higher-than-average for patients with depression. The Web-based exercise might, therefore, be a preferred therapeutic option for patients with depression who were physically active before the manifestation of the depressive disorder.

### Efficacy in Major Depressive Disorder

IG patients showed a significant decline in depressive symptoms after 8 weeks in both self- and clinician rating. Moreover, the intervention led to a significant improvement in self-efficacy and quality-of-life items “emotional well-being” and “social functioning.”

Interestingly, we had already observed a significant IG response within 6-12 days of the commencement of the study. Both QIDS-SR and QIDS-C scores were reduced by ≥50% in 36% (5/14) and 21% (3/14) patients, respectively. An early reduction of ≥20% in depressive symptoms was assessed in 71% (10/14) patients in QIDS-SR scores and in 50% (7/14) patients in QIDS-C scores, which is comparable to the response rate to selective serotonin reuptake inhibitors (78%) within the first 14 days of treatment reported in a previous study [45]. However, the early response rate to selective serotonin reuptake inhibitors, as described by Tadic et al [45], is likely to be overestimated as it was evaluated under conditions of inpatient treatment that entails multimodal therapy with several putative antidepressive factors.

Because it is unlikely that exercise results in physiological adaptations within 2 weeks, an early improvement in depressive symptoms may rather be attributed to the increased self-efficacy and/or further factors such as personal contact, care, motivation, ongoing monitoring, and patient expectations toward a positive effect [21,30], that is, factors that are suggested to contribute to placebo effects. Early antidepressive effects of exercise may be mediated by these placebo effects [52], but they may also be explained by the following: (1) disengaged higher-order functions of the prefrontal cortex to keep unhelpful emotional processes from compromising optimal motor execution (transient hypofrontality hypothesis [53]); (2) the dual-mode theory of affective responses to exercise [54]; or (3) the opponent-process theory of emotion [55]. However, even if placebo effects were mainly responsible for the early positive effects of exercise, this would not devalue exercise as a therapeutic option because placebo effects are likely to constitute a large proportion of the response to antidepressants as well [56].

In contrast to the initial antidepressive effects, the sustained reduction in depressive symptoms after 8 weeks could be attributed to physiological adaptations triggered by exercise. Regular physical activity over several weeks might have provoked a complex interaction of psychological (eg, increased self-efficacy) and neurobiological (eg, increased serotonin synthesis in the brain) adaptations [31]. As the explorative multilevel analysis revealed, an exponential decay model can be expected for the treatment. Thus, both a time-function and the number of training units per time should be included in future analyses to model the trajectories of the QIDS-SR and QIDS-C variables.

### Comparison to Web-Based Cognitive Behavioral Therapy

Computerized CBT (cCBT) is highly accepted [57] and has garnered increasing attention due to its capability to deliver a potentially effective and efficient therapeutic method to large numbers of people with depression. However, independent evaluations of cCBT have failed to prove relevant clinical benefits in depression [15] unless offered in an individually tailored form [16]. In this study, depressive symptoms declined with individualized Web-based exercise therapy. This therapeutic approach has, thus, a similar potential as individually tailored cCBT. However, in contrast to cCBT, it has the advantage of being cognitively little demanding. In this study, 40% of depressive participants showed a Montreal Cognitive Assessment score of <26, indicating mild cognitive impairment. In depression, attentional and executive functions are often affected, which limits the patients’ capability to follow the cognitive demanding instructions of cCBT. Another advantage of exercise is its positive influence on cardiovascular risk factors, the immune system, bone metabolism, and others [58], which helps prevent somatic disorders such as cardiovascular and cerebrovascular diseases and type 2 diabetes mellitus [26], to which patients with chronic depression are more vulnerable than others. However, a Web-based exercise program has the disadvantage that people must be motivated and physically able to perform exercise therapy. This is a particular challenge in depression because people with depression are, on average, physically little active [59,60]. Thus, it is likely that individualized Web-based exercise will be accepted by a subgroup of depressive patients only. However, patients’ acceptability can be enhanced by starting with a lesser number of exercise units (2 instead of 3) with individually adapted moderate intensity and by providing regular motivational feedback.

### Study Limitations

One objective of this study was to evaluate the acceptability of individualized Web-based exercise therapy and its effects on depressive symptoms. The study was, thus, designed as it is appropriate for a feasibility study. Hence, the sample size was rather small, especially for the control group (n=6). Furthermore, 1 participant in the control group showed full remission at T2 from severe depressive symptoms at study entry, entailing a probable overestimation of antidepressive effects in the control group. Thus, comparisons between the intervention and control groups should be considered as little valid.

Another limitation of this study is its duration of 8 weeks. The exercise duration of 8 weeks with constant 3 endurance units per week usually results in an improvement of all physiological performance parameters (VO_2_ peak, lactate threshold, and maximum output in Watt) [61] On the basis of the results reported by Blumenthal et al [21], we expected that patients with MDD would be able to perform constantly 3 endurance exercises per week. However, most of our patients with MDD were only capable of starting with 2 endurance units per week. Thus, our 8-week exercise intervention led merely to a significant increase in the maximum output in Watt but not in VO_2_ peak and lactate threshold. We, hence, recommend performing regular endurance exercise for, at least, 10 weeks to achieve an improvement in all physiological performance parameters in future MDD studies. This is in line with a previous recommendation for the prescription of exercise in MDD [30].

IG participants performed, on average, 75 minutes of weekly endurance exercise over the 8 weeks. This only meets the minimum level of required exercise for both healthy and depressed subjects [9,34,62], which in combination with the short intervention may have led to a nonsignificant improvement of physiological performance parameters. Nonetheless, patients also benefit from a small amount of physical activity [63].

Follow-up data were not obtained in this feasibility study. Hence, data on the long-term effects of our exercise intervention are missing. Follow-up data on the long-term effects of exercise in MDD are limited in literature, but they indicate a sustained benefit on depressive symptoms [64,65], with a significant number of patients continuing exercise on their own [66]. Babyak et al [65] showed that the antidepressive effects of aerobic exercise were sustained over a follow-up period of 6 months; interestingly, patients in the exercise group had also significantly lower relapse rates than those in the medication group during the follow-up.

No study has hitherto examined the effects of a Web-based exercise intervention. Web-based apps show a trend toward nonusage over time, including nonavailability for follow-up [67]. For these reasons and the risk of relapses in depression [68], we strongly recommend including follow-up measurements in future studies.

In sum, this study proved that an individualized Web-based exercise program of 8 weeks is feasible and well accepted. In addition, the exercise program led to a significant and clinically relevant improvement of depressive symptoms in the IG. Expensive performance diagnostics might be dispensable if exercise starts with only 2 endurance units per week of low intensity. Furthermore, exercise recommendations for the upcoming week can then be given on the basis of the performance in the last week (Figure 3) according to a strict algorithm.

Moreover, the coherence between self-rated and clinician-rated depressive symptoms was high. Thus, rating by a physician is redundant. Regular motivational feedback and goal-setting—key factors to high adherence, especially in Web-based settings [12,30,31,38]—can also be generated by a Web-based therapist. As a consequence, rating of depressive symptoms as well as exercise recommendations could be given without the involvement of a physician. If such modifications are implemented, our exercise program is suitable for full computerization.

### Conclusions and Implications for Further Research

We showed for the first time that an individualized, Web-based exercise program is feasible and effective in patients with moderate to severe depression. Our program could be an option for (1) patients who do not respond to or do not want to apply pharmaceuticals or psychotherapy and (2) patients who are motivated and physically able [33] to complete a structured exercise program over several weeks. In addition, the study gives important implications for future randomized, fully computerized, and individually tailored exercise trials in MDD.

## Abbreviations

ANCOVA: analysis of covariance
CBT: cognitive behavioral therapy
GSE: General Self-Efficacy scale
HPA: habitual physical activity
IG: intervention group
MDD: major depressive disorder
QIDS-C: Quick Inventory of Depressive Symptomatology-clinician rating
QIDS-SR: Quick Inventory of Depressive Symptomatology-self-reported

## Appendix

Multimedia Appendix 1 CONSORT‐EHEALTH checklist (V 1.6.1).
